# Acute kidney disease following cisplatin treatment in head and neck cancer: risk factors and survival impact

**DOI:** 10.3389/fmed.2026.1927784

**Published:** 2026-10-14

**Authors:** Yu-Hsiang Hung, Chieh-Ting Chen, Yung-An Tsou, Wei-Chao Chang, Chien-Jen Chiu

**Affiliations:** 1 Department of Otorhinolaryngology–Head and Neck Surgery, China Medical University Hospital, Taichung, Taiwan; 2 School of Medicine, China Medical University, Taichung, Taiwan; 3 Graduate Institute of Biomedical Sciences, China Medical University, Taichung, Taiwan; 4 Department of Otorhinolaryngology–Head and Neck Surgery, Asia University Hospital, Taichung, Taiwan; 5 Center for Molecular Medicine, China Medical University Hospital, Taichung, Taiwan

**Keywords:** acute kidney disease, big data analysis, cisplatin, head and neck squamous cell carcinoma, kaplan–Meier survival curve

## Abstract

**Background:**

Acute kidney disease (AKD), representing persistent or subacute kidney dysfunction occurring within 7 to 90 days, has emerged as an important framework bridging acute kidney injury (AKI) and chronic kidney disease. However, the occurrence and long-term clinical implications of AKD following cisplatin treatment in patients with head and neck squamous cell carcinoma (HNSCC) remain incompletely characterized.

**Methods:**

This study retrospectively analyzed patients with HNSCC treated with cisplatin using a longitudinal institutional clinical database. AKD was classified using the prespecified renal-function criteria applied in the original data-extraction algorithm during the 7–90-day period following cisplatin treatment. Clinical characteristics, AKD occurrence, and 5-year all-cause mortality were evaluated.

**Results:**

Among the 2,033 patients, AKD occurred in 575 (28.3%) patients. Diabetes mellitus was more prevalent among patients with AKD than among those without AKD (20.0% vs. 12.3%, *p* < 0.001) in univariable analysis. In the prespecified Cox model, the association between AKD and 5-year all-cause mortality did not reach statistical significance (adjusted HR, 1.09; 95% CI, 0.94–1.25). Only two ESKD events were observed during follow-up (0.1%).

**Conclusion:**

AKD was observed in 28.3% of this historical cohort of patients with HNSCC treated with cisplatin. Diabetes mellitus was more prevalent among patients with AKD in univariable analysis. The association between AKD and 5-year all-cause mortality did not reach statistical significance in the prespecified adjusted model, although the confidence interval did not exclude a clinically meaningful increase in risk.

## Introduction

Head and neck squamous cell carcinoma (HNSCC) is the fifth most common cancer worldwide, with approximately 946,000 new cases and 485,000 deaths reported annually (1). The treatment options include surgery, radiation therapy, and chemotherapy. The most common chemotherapeutic agents include cisplatin, carboplatin, paclitaxel, docetaxel, and fluorouracil. Cisplatin is used to treat various other cancers, including germ cell tumors (2), cervical cancers (3), lung cancers (4, 5), colorectal cancers (6), and ovarian cancers (7). It can be used in adjuvant therapy or induction therapy and, when combined with surgery and radiation therapy, constitutes a complete curative-intent treatment strategy (8). However, cisplatin resistance remains a major challenge, affecting intrinsically resistant tumors and tumors that acquire resistance after treatment (9). Nephrotoxicity is one of the most recognized adverse effects of cisplatin, with acute kidney injury (AKI) occurring within 7 days and acute kidney disease (AKD) persisting for 7 to 90 days representing the most severe manifestations. This toxicity is directly linked to the mechanism of action of cisplatin. The drug enters cells, including renal epithelial cells, via the organic cation transporters OCT1 and OCT2, where it induces mitochondrial and nuclear DNA damage, ultimately triggering cellular apoptosis (10). Patients with HNSCC are relatively vulnerable to nephrotoxicity while undergoing treatment, when anatomical disruptions and functional impairments (such as xerostomia and dysphagia) can lead to insufficient oral hydration, significantly elevating the risk of treatment-related renal complications (11–13). Among hospitalized patients, AKI occurs in approximately 2%–7%, with 5%–6% requiring renal replacement therapy. The overall hospital mortality rate for patients with AKI is reported to be 60.3% (14, 15). In summary, while the existing literature has identified numerous renal risks associated with HNSCC, there remains a relative scarcity of big data analytics focused on AKD. Particularly in the current era, where ADQI consensus and KDIGO guidelines public-review draft have established AKI and AKD as a clinical continuum, further research into this transitional phase is essential (16, 17). The primary objectives of this study were to evaluate whether AKD followed by cisplatin treatment significantly affects patient survival and to identify the risk factors that increase susceptibility to AKD during chemotherapy (18). Based on the physiological vulnerabilities of HNSCC patients, we hypothesized that the occurrence of AKD significantly correlates with poorer overall survival.

## Methods and management

### Treatment strategy

The National Comprehensive Cancer Network guidelines recommend a cisplatin dose of 100 mg/m^2^ (8). However, because the primary study cohort consisted of Asian individuals, who generally have smaller body sizes and genetic polymorphisms affecting DNA repair mechanisms, the standard cisplatin dose used in the present study was 75 mg/m^2^, infused over 3 h every three weeks for 3 planned cycles; the cumulative dose was 225 mg/m^2^ (19). The study population included patients under curative-intent protocols [post-operation chemoradiation therapy (post-op CRT) and definitive concurrent chemoradiotherapy (CCRT)]. Following clinical standards, the planned cumulative cisplatin dose for these patients was consistently targeted at 200–300 mg/m^2^ (8, 20, 21). If a patient developed clincal significan AKI/AKD or another renal contraindication, a nephrology consultation was initiated for inpatient admission to ensure adequate evaluation and treatment, and an alternative TFH protocol (paclitaxel, fluorouracil, hydroxyurea) was considered.

Patient management included pre-hydration (1,000 mL of 0.9% normal saline over 2 h) and post-hydration (2000mL over 20 h) relative to cisplatin infusion. To mitigate chemotherapy-induced nausea and vomiting (CINV) and prevent subsequent dehydration or electrolyte imbalance, a triple antiemetic regimen consisting of dexamethasone, a 5-HT3 receptor antagonist (ondansetron), and an NK1 receptor antagonist (aprepitant) was administered. Renal function—including serum creatinine, blood urea nitrogen (BUN), eGFR, sodium, potassium, and urine output—was assessed before each cycle. Follow-up blood tests were performed within one week post-treatment in either an inpatient or outpatient setting. Consequently, each patient underwent at least six renal function assessments throughout the three treatment cycles (approximately 50 days), with follow-up clinic visits occurring at least every two weeks (8, 19). For patients with renal contraindications, hospitalization was required for close monitoring, adequate hydration, and avoidance of nephrotoxic medications, such as non-steroidal anti-inflammatory drugs (NSAIDs) and aminoglycosides. Renal function was reassessed after one week to determine whether chemotherapy could be continued.

### Study cohort

In 2020, the China Medical University Hospital (CMUH) established the iHi Data Platform, in which data from various clinical sources were verified and validated to unify traceable patient information generated during healthcare processes. Primary diagnoses of head and neck squamous cell carcinoma of the oral cavity, oropharynx, hypopharynx, and larynx (ICD codes C00–C14, C32) were identified. The medical records of 3,182,935 patients who were provided with care at CMUH between January 1, 2003, and December 31, 2022, were documented in the iHi Data Platform, and detailed information on the iHi Data Platform has been published (22). This retrospective cohort study included 2,716 patients diagnosed with HNSCC who received curative-intent intravenous cisplatin treatment at CMUH between 2004 and 2022. The patient treatment strategies included definitive CCRT and post-op CRT. To minimize confounding factors arising from treatment heterogeneity, patients were excluded if they expired within two weeks after receiving cisplatin, underwent non-curative-intent treatment, had recurrent disease, were kidney transplant recipients, had pre-existing end-stage kidney disease (ESKD) or a history of other cancers prior to initiation of cisplatin treatment, lacked serum creatinine measurements before or three months after treatment, had clinical Stage 0 disease, or were lost to follow-up. This approach avoided misclassifying terminal multi-organ failure as AKD followed by cisplatin treatment but is noted as a potential source of temporal bias in the study limitations. A total of 2,033 cisplatin recipients were included in the analysis (Figure 1). The Charlson Comorbidity Index (CCI) score was calculated for each patient based on the International Classification of Diseases, Ninth or Tenth Revision, Clinical Modification recorded prior to cohort entry, using the coding algorithm proposed by Quan et al. (23). All enrolled patients were followed up until their first diagnosis of ESKD, their death, the date of their last visit, or December 31, 2022, whichever occurred first. This study was approved by the Big Data Center of CMUH and the Research Ethical Committee/Institutional Review Board (REC/IRB) of CMUH (CMUH111-REC2-155 and CMUH113-REC3-033).

**Figure 1:**
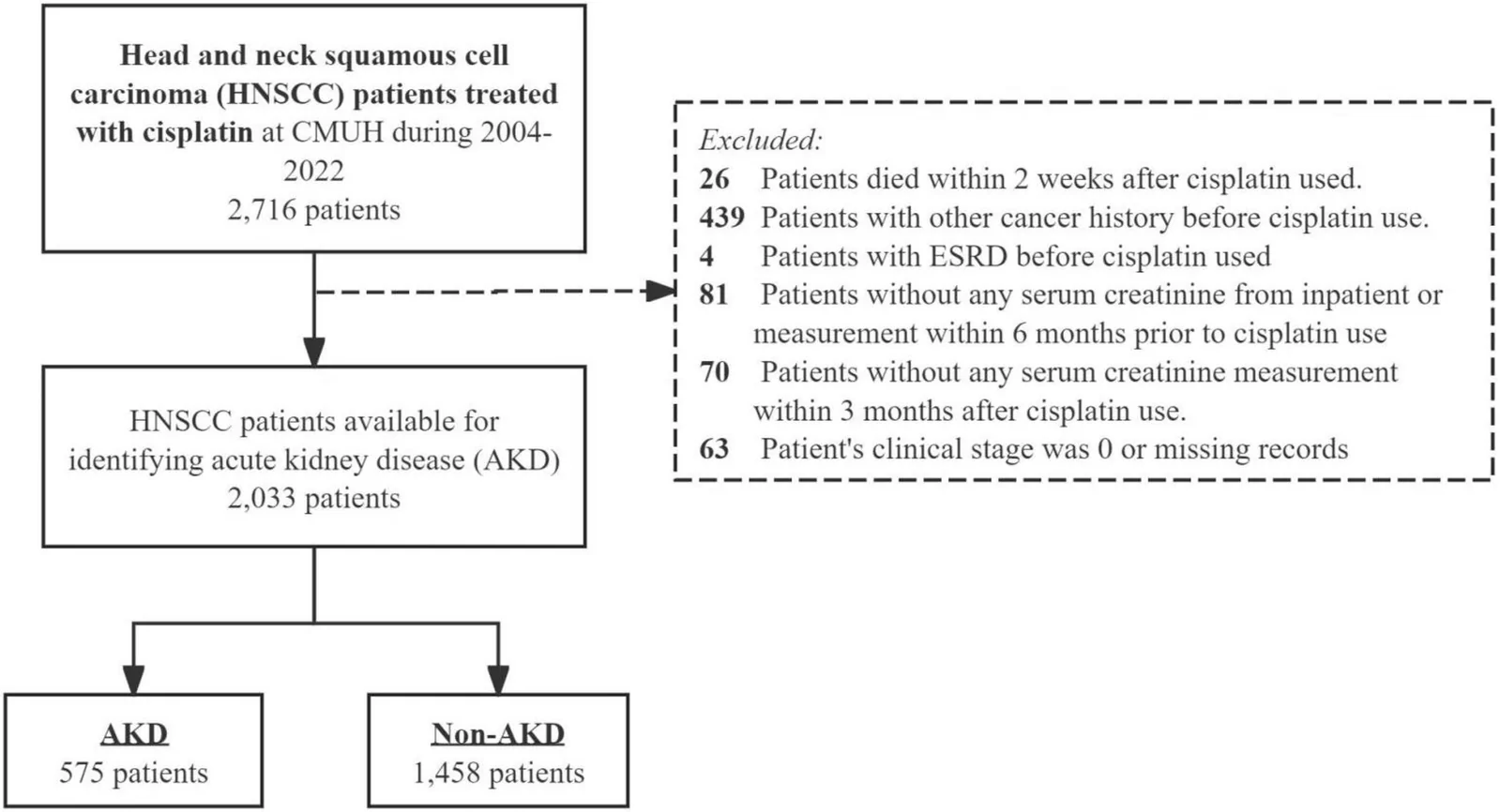
Study cohort selection flowchart.

### Determination of kidney function and ascertainment of outcome

Serum creatinine (SCr) levels were measured at the Central Laboratory of CMUH using the Jaffe rate method, which is based on the kinetic alkaline picrate reaction, using a Beckman UniCel DxC 800 immunoassay system (Beckman Coulter Inc., Brea, CA, USA). The estimated glomerular filtration rate (eGFR) was calculated using the Chronic Kidney Disease Epidemiology Collaboration creatinine equation (24). The baseline SCr level was defined as the closest outpatient measurement obtained within six months prior to cisplatin treatment; if this was unavailable, the nearest scheduled inpatient SCr level before cisplatin treatment was used. The definition of acute kidney disease (AKD) is presented below (18, 25):

A. Meeting AKI criteria and persisting for more than 7 days but less than 90 days
1. Increase in SCr by ≥0.3 mg/dl (≥26.5 µmol/l) within 48 h;
2. Increase in SCr by ≥1.5 times baseline that is known or presumed to have occurred within the prior 7 days;
3. Mean urine volume of less than 0.5 mL/kg/h (based on ideal body weight) for ≥6 h.
B. Meeting AKD criteria and persisting for less than 90 days
1. GFR <60 mL/min per 1.73 m^2^;
2. Decrease in GFR by ≥35 mL/min per 1.73 m^2^ from baseline;
3. Increase in SCr by >50%.

Because renal function after cisplatin treatment was assessed according to routine clinical practice rather than continuous inpatient monitoring, persistence of renal dysfunction was determined from serial laboratory measurements obtained during follow-up. Patients were generally discharged after treatment and underwent repeated renal-function testing during subsequent outpatient or inpatient visits. Persistence beyond 7 days was therefore determined when renal dysfunction remained evident on follow-up measurements beyond the acute 7-day period and within the 90-day AKD window, rather than through uninterrupted daily measurements. Dates of death were ascertained through the National Death Registry maintained by the Ministry of Health and Welfare, Taiwan (26, 27). Patients with ESKD were identified using certificates of catastrophic illness issued by the National Health Insurance Administration. The primary study outcomes were the incidence of ESKD and all-cause mortality.

### Statistical analyses

Continuous variables were analyzed using the Wilcoxon rank-sum test and are presented as medians (interquartile ranges). Categorical variables were analyzed using the chi-square test and are reported as frequencies (percentages). Associations between AKD status and clinical outcomes, including incident all-cause mortality, were assessed using multivariable Cox proportional hazards regression models. ESKD was summarized descriptively because only two events occurred during follow-up (28). Survival differences between patients with and without AKD were further assessed using Kaplan–Meier survival curves, and statistical significance was tested using the log-rank test. A two-tailed significance level (*α*) of 0.05 was used to determine statistical significance. All statistical analyses were performed using SAS (version 9.4; SAS Institute Inc., Cary, NC, USA). Kaplan–Meier curves were generated using R (version 3.5.1; R Foundation for Statistical Computing, Vienna, Austria).

## Results

As illustrated in Table 1, a total of 2,716 patients with head and neck squamous cell carcinoma (HNSCC) who received intravenous cisplatin between 2004 and 2022 were initially identified through the iHi Data Platform at China Medical University Hospital. To ensure methodological transparency and accurate identification of AKD, a total of 683 patients were excluded based on strict criteria.

**Table 1:** Baseline demographic and clinical characteristics of 2,033 patients with head and neck squamous cell carcinoma, with and without acute kidney disease, who were treated with cisplatin during 2004-2022 at China medical university hospital.

Characteristic	Available N	Total *N* = 2,033	Non-AKD *N* = 1,458	AKD *N* = 575	*P*-value^a^
Age, years	2033	53.3 (46.7, 60.0)	53.4 (46.8, 60.3)	52.9 (46.5, 59.6)	0.198
Female	2033	110 (5.41)	76 (5.21)	34 (5.91)	0.516
Cancer clinical stage	2033				0.253
Stage I		76 (3.74)	60 (4.12)	16 (2.78)	
Stage II		211 (10.4)	143 (9.81)	68 (11.8)	
Stage III		302 (14.9)	222 (15.2)	80 (13.9)	
Stage IV		1,444 (71.0)	1,033 (70.9)	411 (71.5)	
Cumulative dose (mg/m^2^)	2033	238.53	300	82.65	<0.001
SCr at baseline^b^, mg/dL	2033	0.88 (0.77, 1.00)	0.90 (0.78, 1.01)	0.86 (0.74, 0.98)	<0.001
eGFR at baseline, mL/min/1.73m^2^	2033	100 (88.4, 109)	99.6 (87.5, 108)	103 (91.9, 111)	<0.001
≥ 70		1,984 (97.6)	1,429 (98.0)	555 (96.5)	
50–70		44 (2.16)	26 (1.78)	18 (3.13)	
Baseline comorbidities^c^					
Charlson Comorbidities index	2033	2 (2, 3)	2 (2, 3)	2 (2, 3)	0.008
Myocardial infarct	2033	15 (0.74)	7 (0.48)	8 (1.39)	0.031
Congestive heart failure	2033	22 (1.08)	11 (0.75)	11 (1.91)	0.023
Peripheral vascular disease	2033	11 (0.54)	7 (0.48)	4 (0.70)	0.551
Cerebrovascular disease	2033	66 (3.25)	51 (3.50)	15 (2.61)	0.308
Dementia	2033	10 (0.49)	8 (0.55)	2 (0.35)	0.560
Chronic pulmonary disease	2033	96 (4.72)	62 (4.25)	34 (5.91)	0.112
Rheumatologic disease	2033	20 (0.98)	15 (1.03)	5 (0.87)	0.743
Peptic ulcer disease	2033	201 (9.89)	142 (9.74)	59 (10.3)	0.723
Liver disease	2033	245 (12.1)	175 (12.0)	70 (12.2)	0.313
Diabetes	2033	295 (14.5)	180 (12.3)	115 (20.0)	<0.001
Hemiplegia or paraplegia	2033	6 (0.30)	6 (0.41)	0 (0)	0.123
Renal disease	2033	18 (0.89)	10 (0.69)	8 (1.39)	0.126
AIDS	2033	3 (0.15)	1 (0.07)	2 (0.35)	0.140
Outcome, until 2022/12/31					
5-year all-cause mortality	2033	891 (43.8)	620 (42.5)	271 (47.1)	0.059
5-year ESKD	2033	2 (0.10)	2 (0.14)	0 (0)	0.374

a Continuous variables, expressed as medians and interquartile ranges (IQRs), were compared using the Wilcoxon rank-sum test. Categorical variables, expressed as frequencies (percentages), were compared using the chi-squared test.

b Defined as the closest outpatient SCr within 6 months before the index date; if unavailable, the nearest scheduled inpatient SCr was used.

c Baseline comorbidity diagnosis within one year before the index date.

AIDS, acquired immunodeficiency syndrome; AKD, acute kidney disease; CKD, chronic kidney disease; eGFR, estimated glomerular filtration rate; ESKD, end-stage kidney disease; SCr, serum creatinine.

The primary exclusions involved 26 patients who expired within 2 weeks (14 days) of cisplatin administration; these were excluded to ensure a complete diagnostic window for AKD and to minimize confounding factors from terminal multi-organ failure. Furthermore, 439 patients with a history of other cancers and 4 patients with pre-existing end-stage kidney disease (ESKD) were removed. To ensure data integrity for renal function assessments, 81 patients without baseline serum creatinine (SCr) measurements (within 6 months prior to treatment) and 70 patients lacking follow-up SCr data (within 3 months post-treatment) were excluded. Finally, 63 patients were excluded due to having clinical Stage 0 disease or missing records.

The final study cohort consisted of 2,033 patients. Following the KDIGO/ADQI criteria—which defines AKD as functional or structural abnormalities persisting for 7 to 90 days—the patients were categorized into two groups: the AKD group (*n* = 575; 28.3%) and the non-AKD group (*n* = 1,458; 71.7%). The cohort included 110 females, with a median age of 53.3 years, and there were no significant differences in age (*p* = 0.198) or sex (*p* = 0.516) between the AKD and non-AKD groups. Most patients were diagnosed with Stage IV HNSCC, and they were distributed as follows: Stage I, 76 patients (3.74%); Stage II, 211 patients (10.4%); Stage III, 302 patients (14.9%); and Stage IV, 1,444 patients (71.0%). All 1,458 patients without AKD completed the three planned cisplatin cycles, corresponding to a cumulative dose of 225 mg/m^2^. Among the 575 patients who developed AKI/AKD, renal dysfunction occurred after the first cycle in 166 patients (28.9%), after the second cycle in 278 patients (48.3%), and after completion of the third cycle in 131 patients (22.8%). Thus, 444 of 575 patients (77.2%) developed renal dysfunction before completion of the planned three-cycle cisplatin regimen. The cisplatin exposure was presented in Table 2. Regarding comorbidities based on the CCI, the prevalence of myocardial infarction (*p* = 0.031), congestive heart failure (*p* = 0.023), and diabetes was more prevalent among patients with AKD than among those without AKD (20.0% vs. 12.3%, *p* < 0.001). Only two ESKD events were observed during follow-up, both in the non-AKD group. Given the very small number of events, ESKD was summarized descriptively and no inferential analysis was performed. The 5-year all-cause mortality rates were 47.1% in the AKD group and 42.5% in the non-AKD group. All-cause mortality was marginally higher in the AKD group and narrowly failed to achieve statistical significance (*p* = 0.059).

**Table 2:** Cisplatin exposure table.

Renal status/timing	n	%	Cumulative cisplatin exposure
No AKD; completed 3 cycles	1,458	100% of non-AKD	225 mg/m^2^
AKI/AKD after cycle 1	166	28.9% of AKD	75 mg/m^2^
AKI/AKD after cycle 2	278	48.3% of AKD	150 mg/m^2^
AKI/AKD after cycle 3	131	22.8% of AKD	225 mg/m^2^

All-cause mortality across the two groups was subsequently analyzed (Table 3). During the five years of follow-up, the mortality rate was 16.1 deaths per 100 person-years in the AKD group and 15.1 deaths per 100 person-years in the non-AKD group. In the Cox proportional hazards model, after adjustments for age, sex, baseline eGFR, CCI, and cancer stage, patients in the AKD group had a 1.09-fold higher risk of death compared to those in the non-AKD group [adjusted hazard ratio [HR], 1.09; 95% confidence interval [CI], 0.94–1.25], which was consistent with the crude model (HR, 1.09; 95% CI, 0.95–1.26).

**Table 3:** Hazard ratio (95% confidence interval) for 5-year all-cause mortality according to acute kidney disease status in patients with head and neck squamous cell carcinoma who were treated with cisplatin.

AKD status	No. of deaths	Person-years	Mortality^a^	HR (95% CI)	
				Crude Model	Adjusted Model
Non-AKD	620	4,097	15.1	1.00 (Reference)	1.00 (Reference)
AKD	271	1,679	16.1	1.09 (0.95–1.26)	1.09 (0.94–1.25)

The model was adjusted for age, sex, baseline estimated glomerular filtration rate, clinical cancer stage, and Charlson Comorbidity Index score.

a per 100 person-years.

AKD, acute kidney disease; CI, confidence interval; HR, hazard ratio.

The Kaplan–Meier curves for 5-year all-cause mortality among patients with HNSCC, with and without AKD, are shown in Figure 2. The log-rank test results indicated no statistically significant difference in the probability of survival between the two groups during the follow-up period (*p* = 0.23).

**Figure 2:**
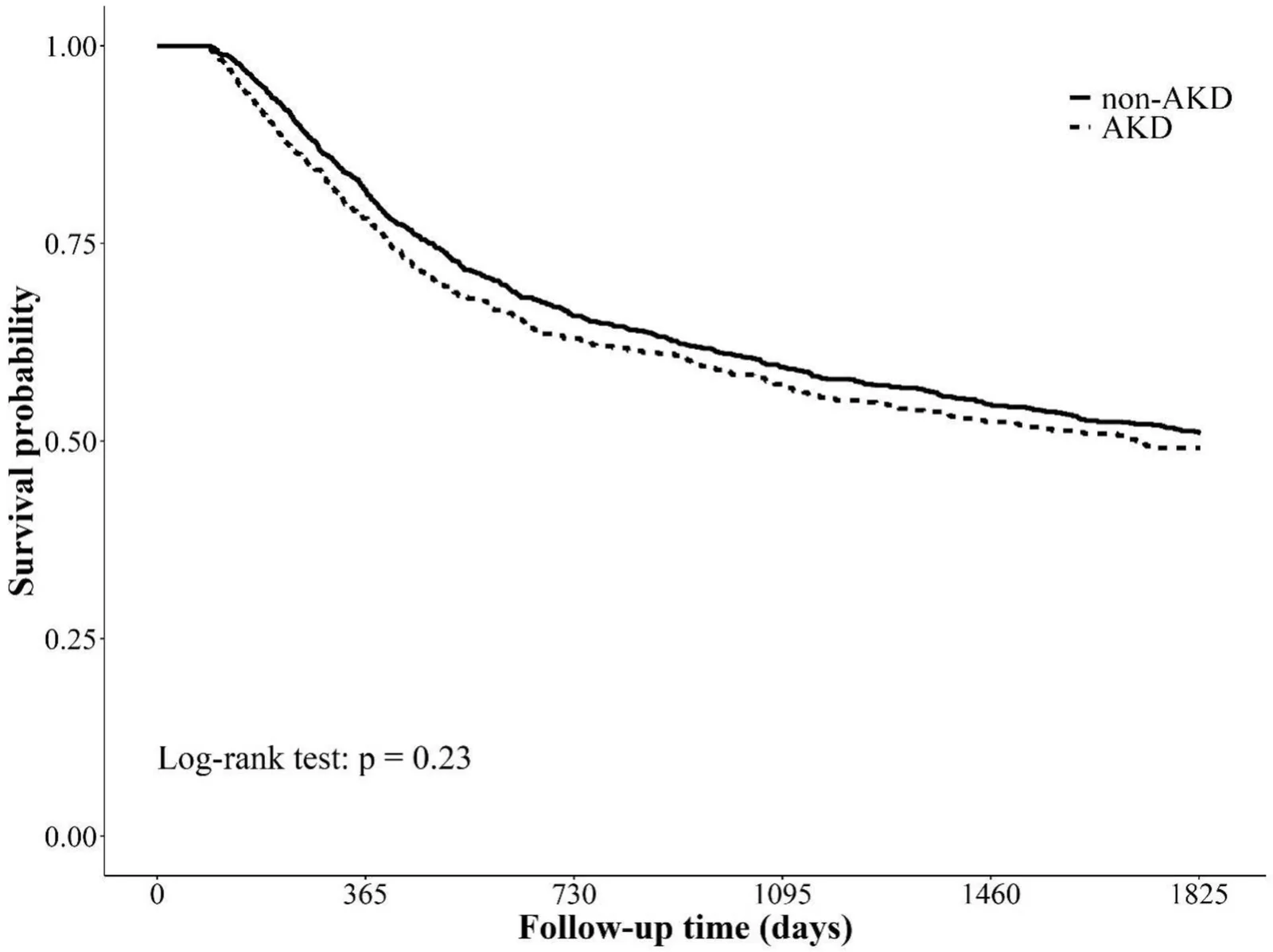
Kaplan–meier survival curves for 5-year all-cause mortality in patients with cisplatin-treated head and neck squamous cell carcinoma, with and without acute kidney disease (AKD). The solid line represents patients without AKD, and the dashed line represents patients with AKD.

## Discussion

In this retrospective cohort of 2,033 patients with HNSCC treated with cisplatin, AKD was observed in 28.3% of patients. DM was more prevalent among patients with AKD in univariable analysis. The association between AKD and 5-year all-cause mortality did not reach statistical significance in the prespecified adjusted Cox model, although the confidence interval remained compatible with a clinically meaningful increase in mortality risk. Only two ESKD events were observed, precluding meaningful inference regarding long-term kidney failure.

Traditionally, the KDIGO guidelines have been the primary standard for analyzing kidney diseases. In the 2012 version, the medical community focused predominantly on acute kidney injury (AKI) (within 7 days) and chronic kidney disease (CKD) (exceeding 90 days) (25). This clinical focus resulted in an 83-day “gray zone”; patients whose renal impairment persisted for more than 7 days but less than 90 days lacked a formal definition in the official classifications of that time. It was not until 2017–2018 that the Acute Disease Quality Initiative (ADQI) proposed the definition of acute kidney disease (AKD) (7 to 90 days) to bridge this gap (18). Although this concept has since been widely cited in numerous studies, it remained an “expert consensus” rather than an official KDIGO guideline. Consequently, the majority of research continued to focus on AKI. The ADQI consensus along with the major 2026 KDIGO update, which, for the first time, formally integrated AKD from a supplementary concept into the official diagnostic framework, recognizing AKI, AKD, and CKD as a continuous spectrum (continuum). By utilizing a Big Data Center approach, our study not only aligns with these latest guidelines but also overcomes the challenges of incomplete documentation and analysis inherent in the long-term follow-up required for AKD research. Returning to our initial hypothesis, although our data did not show a statistically significant difference in mortality based on the presence of AKD, the concept of AKD offers greater clinical flexibility in patient monitoring. For HNSCC patients, who require regular follow-up visits rather than long-term hospitalization, AKD serves as a more suitable indicator of renal recovery compared to the hyper-acute parameters of AKI.

Cisplatin, a widely used chemotherapeutic agent, is filtered by the glomeruli and accumulates in the proximal convoluted tubules. Previous studies have shown that the concentration of cisplatin in the renal cortex is approximately five times higher than that in the bloodstream, which leads to renal tubular toxicity (29). The risk of intrinsic ARF associated with cisplatin, an essential drug for the treatment of head and neck cancers, has long been established. Previous studies have reported that approximately 30.5% of patients develop AKI following cisplatin treatment (30–32). In the present study, the observed incidence of AKD was 31.7%. This comparable rate suggests that, while initial renal insults often manifest as AKI within the first week, our expanded 90-day AKD framework successfully captured these events while providing a more comprehensive view of the renal recovery continuum.

Latcha et al. studied the relationship between kidney damage and cisplatin treatment. They monitored the eGFR to assess kidney function and disease progression. Their results indicated that, although cisplatin-induced kidney damage is generally mild and self-limiting, it can be irreversible. A five-year follow-up analysis of CKD revealed that patients with Stage 1 CKD had a 70% likelihood of progressing to a more advanced stage, whereas those with Stage 2 or higher CKD generally remained at their original stage. During the follow-up period, only a small percentage of patients (<5%) developed end-stage kidney disease (ESKD), and none required renal replacement therapy (32). Other studies have also supported this view (33). In the present study, which included 2,033 patients with 575 recorded AKD event, the outcomes were similar, with no patients requiring emergency hemodialysis and only two progressing to ESKD (0.1%). Although patients with DM are more likely to be susceptible to cisplatin-induced AKD, our findings suggest that the HR for mortality did not significantly differ between the AKD and non-AKD groups, under appropriate indications, management and stringent monitoring. These patients can start with standard cisplatin-based treatment. Maurice et al. conducted a statistical analysis of the risk factors of cisplatin-induced AKI in patients with head and neck cancer. Their results indicated that hypertension and uncontrolled chemotherapy-induced nausea and vomiting are significant risk factors, and there were no differences in sex and age between the groups (34). Similarly, in the present study, there were no significant differences between the AKD and non-AKD groups in terms of age, sex, or cancer stage. We also examined whether cardiovascular diseases, including myocardial infarction, congestive heart failure, and peripheral vascular disease, were associated with cisplatin-induced AKD; however, there were no significant differences between the two groups. This lack of association may be attributed to the limited statistical power due to the small number of patients with these comorbidities. In contrast, patients with diabetes was more prevalent among patients with AKD than among those without AKD (20.0% vs. 12.3%, *p* < 0.001), a finding that has not been widely reported in previous studies. Although this univariable association identifies diabetes as a prominent potential risk factor, it aligns with the biological plausibility of renal microvascular complications. It is well established that hyperglycemia stimulates mesangial cells to synthesize angiotensin II, leading to increased glomerular capillary pressure and enhanced vascular permeability (35–37). Immunologically, hyperglycemia promotes macrophage infiltration and inflammation while also inducing oxidative stress, which contributes to long-term vascular damage and hemodynamic abnormalities in the kidneys (38–40). Considering the aforementioned risk factors, the additional stress caused by cisplatin, which affects the renal tubular epithelium and vasculature, makes the occurrence of AKD theoretically plausible. Therefore, in addition to managing nephrotoxicity with appropriate medications, patients receiving cisplatin treatment may require stricter blood glucose control and dietary management. Further studies are required to confirm these findings. Patients with end-stage kidney disease (ESKD) typically require intensive care, frequent monitoring, and regular hemodialysis. In our study, only two ESKD events were observed during follow-up, precluding meaningful inference regarding progression to long-term kidney failure. This low event rate precluded an effective or meaningful analysis regarding the underlying causes or specific management strategies for this population.

This study has several limitations. First, its retrospective, single-center design and reliance on a historical institutional database inevitably introduce the possibility of missing data, residual confounding, and misclassification. Although the longitudinal data platform facilitated assessment of renal function in a large cohort, detailed patient-level information on actual delivered cisplatin dose intensity, dose modifications, treatment delays, concomitant nephrotoxic medications, hydration adherence, and other treatment-related factors was incomplete. Treatment decisions may also have been influenced by patients’ underlying clinical conditions, resulting in potential confounding by indication. In addition, although AKD was classified using the prespecified renal-function criteria of the original data-extraction algorithm. Because renal-function testing was performed at clinically determined intervals rather than continuously, the exact onset and duration of renal dysfunction could not always be determined precisely, introducing the possibility of temporal outcome misclassification.

Second, several limitations specifically affect interpretation of the survival analysis. Patients who died within 14 days after cisplatin administration were excluded, potentially introducing selection bias, and AKD was modeled as a fixed rather than time-dependent exposure because the exact date of AKD onset could not be reliably reconstructed. Accordingly, immortal-time and other time-dependent biases cannot be excluded. Moreover, the absence of a dedicated multivariable model for AKD predictors means that observed associations, including that with diabetes, should be considered exploratory rather than independent effects. Only two ESKD events occurred, precluding meaningful inference regarding long-term kidney failure. Finally, changes in oncologic and supportive care over the 2004–2022 study period, together with the predominantly Han Chinese population and institutional cisplatin regimen of 75 mg/m^2^ every three weeks, may limit generalizability to other populations, treatment eras, and cisplatin regimens. Prospective multicenter studies with detailed longitudinal treatment and renal-function data are warranted.

## Conclusion

In this retrospective single-center cohort of patients with HNSCC treated with cisplatin, AKD was observed in 28.3% of patients. DM was more prevalent among patients with AKD in univariable analysis, although its independent association with AKD could not be established. The association between AKD and 5-year all-cause mortality did not reach statistical significance in the prespecified adjusted model; however, clinically meaningful excess mortality cannot be excluded. ESKD was infrequently observed, but the small number of events precluded meaningful inference regarding long-term kidney failure. These findings support careful renal monitoring during cisplatin treatment and should be interpreted in light of the limitations inherent to the historical retrospective dataset. Further prospective, multicenter studies incorporating detailed cisplatin exposure and time-dependent renal-function data are warranted.

## Data Availability

The raw data that support the findings of this study are available from CMUH, but restrictions apply to their availability. The data were used under license for the current study and are not publicly available; however, they are available from the authors upon reasonable request and with the permission of CMUH.
